# Risk of fracture according to temporal changes of low body weight changes in adults over 40 years: a nationwide population-based cohort study

**DOI:** 10.1186/s12889-023-15940-0

**Published:** 2023-05-25

**Authors:** Jung Guel Kim, Jae-Young Hong, Jiwon Park, Sang-Min Park, Kyungdo Han, Ho-Joong Kim, Jin S. Yeom

**Affiliations:** 1 Spine Center and Department of Orthopaedic Surgery, Seoul National University College of Medicine and Seoul National University Bundang Hospital, 82, Gumi-Ro 173 Beon-Gil, Bundang-Gu, Seongnam-Si, Gyeonggi-Do 13620 Republic of Korea; 2 Department of Orthopedics, Korea University Hospital, Ansan, 123, Jeokgeum-Ro, Danwon-Gu, Ansan-Si, Gyeonggi-Do 15355 Republic of Korea; 3 Department of Statistics and Actuarial Science, Soongsil University, 369 Sangdo-Ro, Dongjak-Gu, Seoul, 06978 Republic of Korea

**Keywords:** Body mass index, Low body weight, Fracture, Risk factors, Temporal changes

## Abstract

**Background:**

Low body weight is associated with an increased risk of fractures. However, the effect of temporal changes in the low body weight status on the risk of fracture remains unknown. This study aimed to evaluate the relationships between temporal changes in low body weight status and the risk of fractures in adults over the age of 40 years.

**Methods:**

This study included data on adults over 40 years old who underwent two biannual consecutive general health examinations between January 1, 2007 and December 31, 2009 extracted from the National Health Insurance Database, a large nationwide population database. Fracture cases in this cohort were monitored from the time of the last health examination to the end of the designated follow-up period (from January 1, 2010 to December 31, 2018) or the participant's death. Fractures were defined as any fracture resulting in hospitalization or outpatient treatment claim after the date of general health screening. The study population was then separated into four groups based on the temporal changes in low body weight status as follows: low body weight to low body weight (L-to-L), low body weight to non-low body weight (L-to-N), non-low body weight to low body weight (N-to-L), and non-low body weight to non-low body weight (N-to-N). The hazard ratios (HRs) for new fractures, depending on weight changes over time, were calculated using Cox proportional hazard analysis.

**Results:**

Adults in the L-to-L, N-to-L, and L-to-N groups had a substantially increased risk of fractures after multivariate adjustment (HR, 1.165; 95% confidence interval [CI], 1.113–1.218; HR, 1.193; 95% CI, 1.131–1.259; and HR, 1.114; 95% CI, 1.050–1.183, respectively). Although the adjusted HR was greater in participants who changed into having a low body weight, followed by those with consistently low body weight, those with low body weight remained to have an elevated risk of fracture independent of weight fluctuation. Elderly men (aged over 65 years), high blood pressure, and chronic kidney disease were significantly associated with an increase in fractures (*p* < 0.05).

**Conclusion:**

Individuals aged over 40 years with low body weight, even after regaining normal weight, had an increased risk of fracture. Moreover, having a low body weight after having a normal body weight increased the risk of fractures the most, followed by those with consistently low body weight.

**Supplementary Information:**

The online version contains supplementary material available at 10.1186/s12889-023-15940-0.

## Introduction

Fractures are one of the main causes of morbidity and mortality in adults, especially in older individuals [1]. Fractures are also strongly associated with higher social expenses as they can result in prolonged absences from work, extensive use of medical resources, and long-term disability [2, 3]. This can lead to greater costs for society. There are many well-known risk factors that have been linked to an increased occurrence of fractures [4–10]. Risk factors include age, sex, menopause, being underweight, obesity, smoking, excessive alcohol consumption, and lack of physical exercise. There is a correlation between weight loss and osteoporosis and sarcopenia, and it has been shown that gaining weight can assist in the preservation of bone density [11, 12]. As a result, a reduction in body mass may affect bone density and increase the risk of fractures, whereas an increase in body mass stabilizes bone density and lowers the number of fractures that occur.

Weight is a significant predictor of health status, including metabolic, immunological, reproductive, and musculoskeletal functioning [13]. Low body mass index (BMI) can result in poor physical health, which is directly associated to an increased risk of mortality and morbidity [14, 15]. A low BMI may also be associated with decreased bone density, soft tissue loss, and muscular weakness, thereby increasing the risk of fractures [12]. However, without an increase in muscle mass, weight gain did not prevent fractures but rather increased their occurrence [16]. Owing to adverse views and discrimination against overweight, a larger percentage of individuals, particularly women, are underweight in contemporary culture [17]. According to a recent study, weight gain, weight loss, and intentional weight loss are associated with an increased incidence of fractures; however, associations differ according to fracture location [18]. Weight loss is associated with an increased risk of fractures in the upper and lower limbs and central body, but intentional weight loss is associated with a decreased risk of hip fractures. However, there have been no studies on whether weight change from an underweight status affects the risk of fractures. Using data from the population-based, nationwide Korean National Health Insurance Service (KNHIS) database, we previously studied the relationship between changes in underweight status and hip fractures [6]. The risk of hip fracture was the highest in those who became underweight from having a normal weight, followed by those who remained underweight. However, we did not analyze other fractures. This study aimed to evaluate the relationships between temporal changes in low body weight status and the risk of all types of fractures using a large nationwide population database of adults aged over 40 years from the National Health Insurance Database.

## Methods

### Data source, study design and population

The study protocol was approved by the Institutional Review Board of Korea University Ansan Hospital (approval no. K2021-2601–001). The ethics committees of Korea University Ansan Hospital have waived the requirement to obtain informed consent as the register data analysed in this study are in anonymised and deidentified format. This study was performed in accordance with the tenets of the Declaration of Helsinki, and all research methods were carried out in accordance with appropriate regulations and guidelines.

The KNHIS database includes diagnoses (ICD-10), prescriptions, and procedures for the entire Korean population (approximately 50 million people) [19]. Every 2 years, all Koreans aged over 40 years were checked for their general health [20]. These health screening records comprise anthropometric measures and lifestyle surveys, socioeconomic data, medication and hospitalization data, outpatient data, and death records for the registered Korean population.

**Fig. 1:**
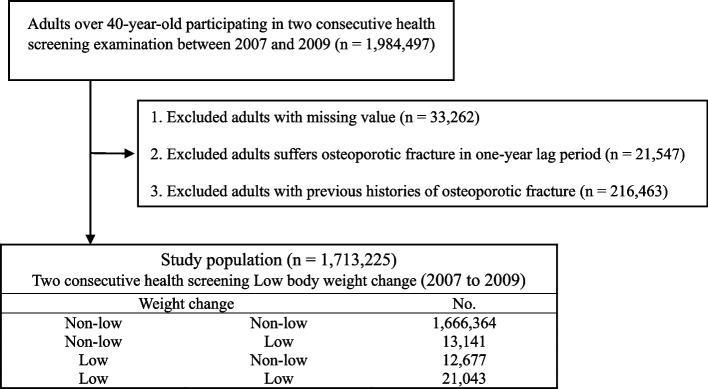
Flow chart of the study population

This database was used to collect information on persons over the age of 40 who underwent two biannual consecutive general health examinations between January 1, 2007 and December 31, 2009, to build a long-term cohort study. Patients with insufficient information and a history of osteoporotic fractures were excluded from the study. The impact of low body weight was magnified by introducing a 1-year delay following the screening procedure. This study included 1,713,225 individuals in total (Fig. 1). Fracture cases in this cohort were monitored from the time of the last health examination to the end of the designated follow-up period (from January 1, 2010 to December 31, 2018) or the participant's death. Fractures were defined as any fracture resulting in hospitalization or outpatient treatment claim after the date of general health screening.

### Evaluation of body weight

This information was derived from the results of the general health examination. BMI was calculated using the following formula: weight in kilograms (kg) divided by height in meters squared (kg/m^2^). Underweight (< 18.5 kg/m^2^), overweight (> 23 kg/m^2^), obesity (> 25.0 kg/m^2^), and severe obesity (> 30.0 kg/m^2^) were categorized according to WHO Asia–Pacific regional recommendations [21, 22].

During each health examination, the patient's body weight status was recorded. In two examinations (2007 and 2009), we determined whether the participants had a low body weight based on BMI measurements. The study population was then separated into four groups based on the temporal changes in low body weight status: low body weight to low body weight (L-to-L), low body weight to non-low body weight (L-to-N), non-low body weight to low body weight (N-to-L), and non-low body weight to non-low body weight (N-to-N).

### Operational definitions of fractures

We used ICD-10 codes, including diagnostic, procedural, and radiographic codes, to search the insurance claims database for all fracture patients [1, 23, 24]. The ICD-10 codes for each fracture were as follows: vertebral fractures (S22.0, S22.1, S32.0, S32.7, T080, M48.4), hip fractures (S72.0 and S72.1), humerus fractures (S42.2, S42.3), and radius fractures (S52.5 and S52.6) [23].

### Covariates and measurements

In this study, the definition of the baseline demographic data was based on the last health examination. These baseline characteristics included socioeconomic data; laboratory results (cholesterol, fasting glucose, blood pressure, and triglyceride); responses to lifestyle questionnaires (regular exercise, smoking, alcohol consumption); anthropometric measurements (height, weight, waist circumference); and medical histories, including hypertension, diabetes, dyslipidemia, and chronic kidney disease (CKD) [25, 26]. Regarding medical history, comorbidities were reported if a health examination or previous medical claim data indicated their presence.

Smoking status was differentiated into non-smokers, former smokers, and current smokers. Based on the amount of alcohol consumed daily, individuals were classified as non-drinkers, light drinkers (less than 30 g/day), or heavy drinkers (> 30 g/day). Regular exercise was defined as 20-min of severe activity at least three times per week or 30 min of moderate to intense exercise at least five times per week. Income was classified as low if it fell within the bottom 20% of the annual income distribution and as normal otherwise. The ICD-10 codes used in this study are listed in Additional file 1.

### Statistical analysis

Baseline data of the study population are presented as means (± standard deviations) or counts (percentages) according to the total number of patients with low body weight. The incidence rate (IR) per 1,000 person-years was used to define the incidence rate (PY). The risk of fracture is presented as hazard ratios (HRs) with 95% confidence intervals (95% CIs) based on Cox regression analysis. To reduce covariate bias, we compared HRs for unadjusted and adjusted models: Model 1 was adjusted for age and sex; Model 2 was adjusted for age, sex, and additional environmental factors, such as smoking, alcohol intake, regular exercise, and low income; and Model 3 was fully adjusted for age, sex, additional environmental factors (smoking, alcohol consumption, and regular exercise), and comorbidities (diabetes, hypertension, dyslipidemia, and CKD). The cumulative incidence was calculated using the Kaplan–Meier technique. Subgroup analysis was conducted using several factors, including age (65 years and > 65 years), sex, regular exercise, low income, and comorbidities. SAS software version 9.3 (SAS Institute, Cary, NC, USA) was used for the statistical analysis. Statistical analyses were performed using the chi-squared test for categorical variables and analysis of variance (ANOVA) for continuous variables. To account for the issue of multiple comparisons, a post-hoc analysis was conducted using the Bonferroni correction method based on the outcomes obtained from the ANOVA and Chi-Square tests. and statistical significance was set at *p* < 0.05 (two-sided).

## Results

### Baseline characteristics

Table 1 provides a summary of the baseline characteristics according to temporal changes in participants with low body weight at each health examination. Based on the change in low body weight status, the 1,713,225 individuals were separated into four groups: N-to-N (1,666,364 persons), N-to-L (12,677 persons), L-to-N (13,141 persons), and L-to-L (21,043 persons). The four groups of N-to-N, N-to-L, L-to-N, and L-to-L individuals indicated statistically significant differences in all categories investigated. The group with the highest incidence of fracture was the N-to-L group (1,353 fractures, 10.3%), followed by the L-to-L group (1,937 fractures, 9.2%), L-to-N group (1,097 fractures, 8.7%), and N-to-N group (125,834 fractures, 7.6%).

**Table 1:** Baseline characteristics of this study according to the body weight changes

Variables	Body weight changes from 2007 to 2009				*p*-value
	N to N	N to L^a^	L to N	L to L	
Participants (n)	1,666,364	13,141	12,677	21,043	
Age (years)	55.19 ± 9.7	57.65 ± 11.93	55.09 ± 11.38	56.08 ± 11.78	< .001
Age (n)					< .001
< 65	1,365,976(81.97)	9,286(70.66)	9,920(78.25)	15,728(74.74)	
≥ 65	300,388(18.03)	3,855(29.34)	2,757(21.75)	5,315(25.26)	
Sex (n)					< .001
Men	876,672(52.61)	5,726(43.57)	5,604(44.21)	9,861(46.86)	
Women	789,692(47.39)	7,415(56.43)	7,073(55.79)	11,182(53.14)	
Height (cm)	162.18 ± 8.71	160.81 ± 8.45	160.98 ± 8.57	161.73 ± 8.3	< .001
Weight (kg)	63.74 ± 10.29	46.39 ± 5.19	50.7 ± 6.32	45.64 ± 5.24	< .001
Smoking (n)					< .001
Non	1,040,628(62.45)	8,818(67.1)	8,341(65.8)	13,458(63.95)	
Ex	311,799(18.71)	1,399(10.65)	1,634(12.89)	2,304(10.95)	
Current	313,937(18.84)	2,924(22.25)	2,702(21.31)	5,281(25.1)	
Alcohol consumption(n)^b^					< .001
Non	953,558(57.22)	8,837(67.25)	8,231(64.93)	13,933(66.21)	
Mild to moderate	605,840(36.36)	3,675(27.97)	3,887(30.66)	6,176(29.35)	
Heavy	106,966(6.42)	629(4.79)	559(4.41)	934(4.44)	
Regular exercise (n)^c^	374,781(22.49)	2,222(16.91)	2,031(16.02)	3,210(15.25)	< .001
Low income (n)^d^	318,842(19.13)	2,744(20.88)	2,668(21.05)	4,274(20.31)	< .001
Comorbidities					
DM (n)	202,779(12.17)	1,130(8.6)	761(6)	1,195(5.68)	< .001
Hypertension (n)	602,227(36.14)	3,145(23.93)	2,510(19.8)	3,725(17.7)	< .001
Dyslipidemia (n)	427,031(25.63)	1,746(13.29)	1,737(13.7)	2,251(10.7)	< .001
CKD (n)	100,980(6.06)	817(6.22)	617(4.87)	1,011(4.8)	< .001
Laboratory findings					
BMI (kg/m^2^)	24.15 ± 2.84	17.89 ± 0.62	19.52 ± 1.5	17.4 ± 0.84	< .001
WC (cm)	81.53 ± 8.31	68.68 ± 6.13	71.06 ± 6.32	66.8 ± 5.51	< .001
Systolic BP (mmHg)	124 ± 14.87	118.37 ± 15.65	118.73 ± 15.36	116.81 ± 15.52	< .001
Diastolic BP (mmHg)	77.05 ± 9.9	73.68 ± 10.1	73.91 ± 9.92	73 ± 9.95	< .001
Fasting glucose	99.93 ± 23.83	96.37 ± 27.21	94.57 ± 20.41	94.19 ± 21.97	< .001
Total cholesterol (mg/dL)	198.01 ± 36.97	187.09 ± 34.34	191.86 ± 34.92	187.19 ± 33.63	< .001
HDL	54.53 ± 22.28	61.67 ± 19.33	60.47 ± 15.12	63.14 ± 17.55	< .001
LDL	117.69 ± 45.79	107.01 ± 39.1	112.03 ± 48.57	106.36 ± 38.34	< .001
eGFR (ml/min/1.73m^2^)	86.86 ± 34.16	89.76 ± 36.31	90.52 ± 31.44	91 ± 35.72	< .001
TG	115.58(115.49–115.68)	83.99(83.32–84.68)	90.33(89.57–91.1)	81.46(80.96–81.95)	< .001

Numeric parameters are expressed as mean ± standard deviation and categorical parameters are expressed as counts and percentages in parentheses

*DM* Diabetes mellitus, *CKD* Chronic kidney disease, *BMI* Body mass index, *WC* Waist circumference, *BP* Blood pressure, *HDL* High-density lipoprotein, *LDL* Low-density lipoprotein, *eGFR* Estimated glomerular filtration rate, *TG* Triglyceride

^a^Low body weight was defined as body mass index under 18.5 kg/m^2^

^b^Alcohol consumption was divided into 3 categories; Non (no alcohol consumption), Mild (under 30 g/day consumption), and heavy (over 30 g/day consumption)

^c^Regular exercise is defined as performing over 30 min moderate intensity exercise over 5 times per a week or over 20 min vigorous intensity exercise over 3 times per a week

^d^Low income is defined as total household monthly income belongs to lower 20% group among Korean entire population

### Risk of fracture according to temporal trends in body mass index changes

The IR was 12.38/1000 PY in the N-to-N group, 17.72/1000 PY in the N-to-L group, 14.46/1000 PY in the L-to-N group, and 15.63/1000 PY in the L-to-L group. Participants in the L-to-L, N-to-L, and L-to-N groups had a substantially increased risk of fractures after multivariate adjustment when comparing to N-to-N group (HR, 1.165; 95% CI, 1.113–1.218; HR, 1.193; 95% CI, 1.131–1.259; and HR, 1.114; 95% CI, 1.050–1.183], respectively). Although the adjusted HR was greater in people changing to having a low body weight, followed by those with consistently low body weight, those with low body weight had an elevated risk of fracture independent of weight fluctuation (Table 2).

**Table 2:** The risk of fracture according to temporal changes in body mass index changes using Cox regression analysis

Low body weight changes^a^	No. of fracture	IR^b^	Unadjusted			Model 1			Model 2			Model 3		
			HR	95% CI	*p*-Value	HR	95% CI	*p*-Value	HR	95% CI	*p*-Value	HR	95% CI	*p*-Value
N to N	125,834	12.38	1		< 0.0001	1		< 0.0001	1		< 0.0001	1		< 0.0001
N to L	1,353	17.72	1.435	1.360 – 1.514		1.202	1.139 – 1.268		1.187	1.125 – 1.252		1.193	1.131 – 1.259	
L to N	1,097	14.46	1.168	1.101 – 1.240		1.118	1.053 – 1.186		1.107	1.044 – 1.175		1.114	1.050 – 1.183	
L to L	1,937	15.63	1.264	1.208 – 1.322		1.171	1.120 – 1.225		1.156	1.105 – 1.209		1.165	1.113 – 1.218	

Model 1 was adjusted by age, and sex

Model 2 was adjusted by age, sex, and other environmental factors such as smoking status, alcohol consumption, regular exercise, low income

Model 3 was fully adjusted by age, sex, other environmental factors (smoking status, alcohol consumption, regular exercise, low income), and comorbidities (diabetes, hypertension, dyslipidemia, chronic kidney disease)

*No* Number, *IR* Incidence rate, *HR* Hazard ratio, *95% CI* 95% confidence interval, *N* Non-low body weight (body mass index ≥ 18.5 kg/m^2^), *L* Low body weight (body mass index < 18.5 kg/m^2^)

^a^Temporal changes of low body weight status (first to 3^rd^ health screening) are divided into four groups: non-low body weight to non-low body weight, non-low body weight to low body weight, low body weight to non-low body weight, and low body weight to low body weight

^b^Incidence rate is defined as incidence rate per 1,000 person-year

**Fig. 2:**
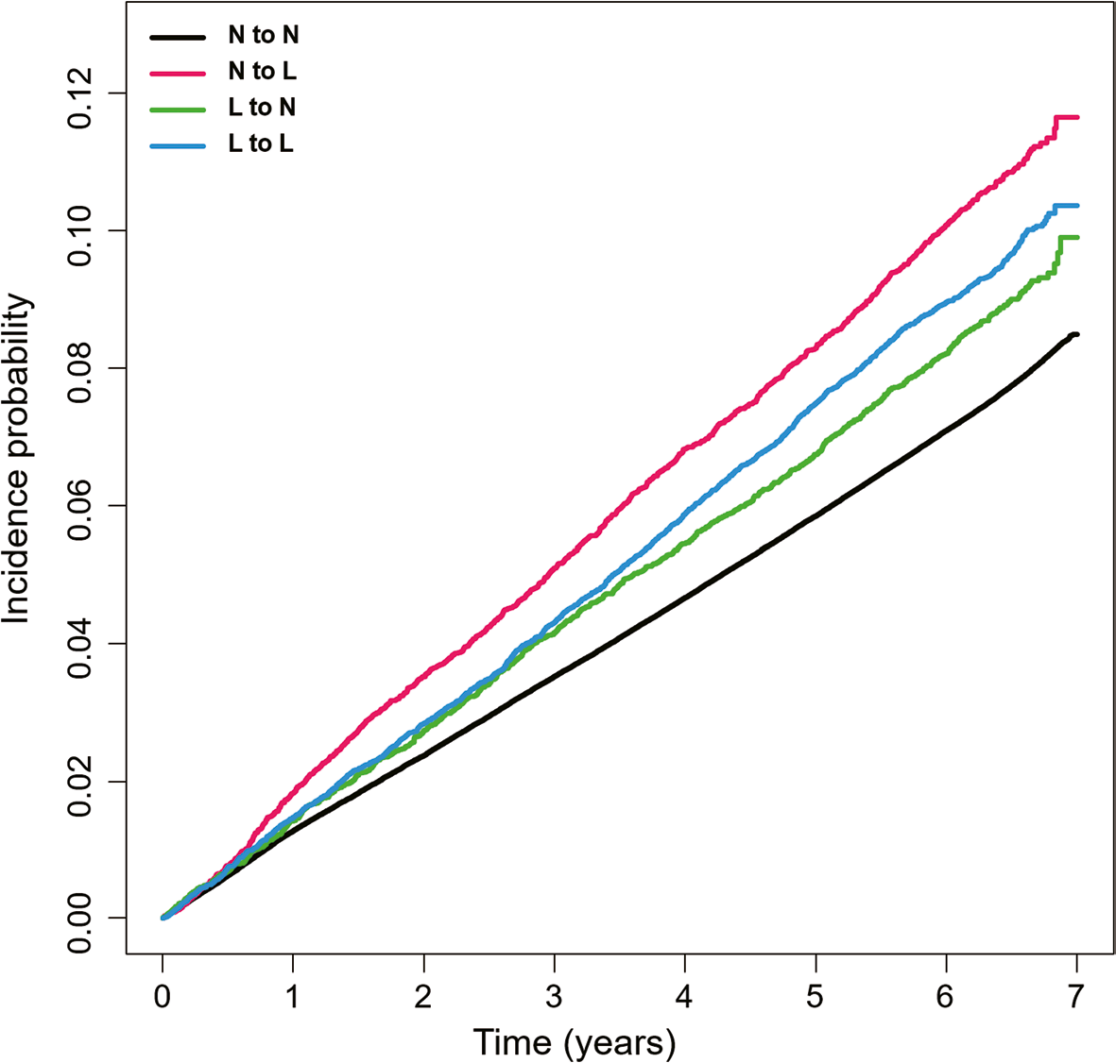
Estimates of the cumulative incidence of fracture according to the temporal changes in the low body weight status

At all time periods, the N-to-L group had a considerably greater cumulative fracture incidence than the other groups, followed by the L-to-L, L-to-N, and N-to-N groups (Fig. 2).

### Subgroup analysis

**Fig. 3:**
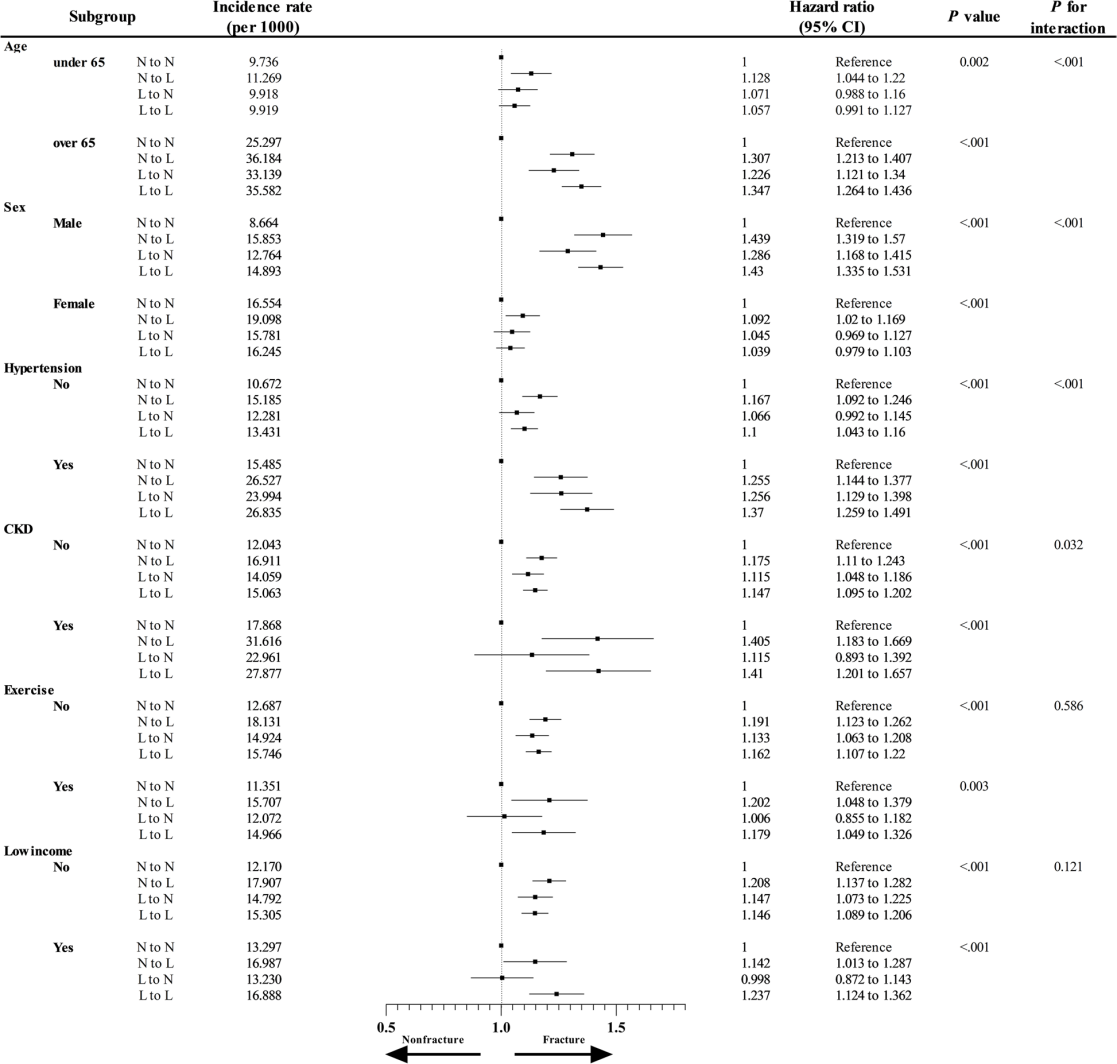
Incidence and hazard ratios of fractures according to several subgroups and the temporal changes in the low body weight status

Fracture risk was adjusted according to BMI and frequency of individuals with low body weight in each subgroup. In subgroups stratified by age, sex, hypertension, CKD, regular exercise, and income, the effect of low body weight on fracture risk was stronger (*p* < 0.05) (Fig. 3). However, depending on the subgroup, the increase in fracture risk associated with temporal changes in low body weight differed. These significant trends were observed in the age, sex, hypertension, and CKD subgroups. The risk was further increased in patients over 65 years of age, in men, and in the presence of hypertension and CKD.

## Discussion

To the best of our knowledge, this is the first general population-based cohort study to determine the risk of fractures associated with temporal changes in the low body weight status. This study found that low body weight increases the risk of fractures in adults aged over 40 years. In particular, changing to having a low body weight mostly increased the risk of fractures, followed by having a consistently low body weight. Furthermore, the risk of fractures was influenced by older age over 65 years, the male sex, hypertension, and CKD.

Our previous investigation identified low body weight as a risk factor for increased fractures [5]; however, the mechanism by which low body weight increases the frequency of fractures remains unclear. We hypothesized that malnutrition is usually associated with being underweight in humans, thereby inducing osteoporosis [27, 28]. Malnutrition can cause bone degeneration and osteoporosis. Additionally, low body weight is strongly associated with the development of sarcopenia. A previous study showed that malnourished individuals are more prone to sarcopenia [29]. Sarcopenia reduces physical strength and muscle function, resulting in injuries that increase the likelihood of fracture [30, 31]. Consequently, a reduced BMI is associated with lower BMD and poorer muscular strength. Because this was a population-based cohort study using ICD-10 diagnosis, procedural, and radiographic codes, real skeletal muscle index and BMD values were unavailable. Although this study cannot definitively explain the relationship between low body weight, BMD, and skeletal muscle index, a large population database has indicated that low BMI is related to fractures.

In the present study, the risk of fracture was 1.14 times higher in the L-to-N group than in the N-to-N group. When body weight is restored to a non-low body weight status, there is an increase in fat mass relative to muscle mass [32, 33]. This may result in the buildup of unhealthy fat, which can further diminish bone mass and strength [34, 35]. In addition, the N-to-L and L-to-L groups in our study had a considerably greater risk of fracture than the N-to-N and L-to-N groups. Furthermore, low body weight from non-underweight status was the main risk factor for weight changes in this study. The explanation for this is as follows. First, increased soft tissue cushioning in the lower extremities may mitigate the damage [18]. Therefore, individuals with low body weight are more susceptible to fractures owing to a lack of fat cushioning. Second, having a low body weight reduces the mechanical demand for weight-bearing, which may influence bone remodeling [36]. Third, being underweight may be linked to lower food intake, especially calcium and protein. Calcium deficiency can diminish BMD, and protein insufficiency can limit the generation of insulin-like growth factor 1, thereby disrupting the bone remodeling process [37, 38]. Adults who changed to having a low body weight were considered important in this study. These are adults who have changed from non-low body weight to low body weight, and it can be considered that muscle and bone loss continuously occur. Adults with consistently low body weight are thought to have a lower risk of fracture than those who have changed to low body weight because the body adapts to low body weight. In our investigation, the link between temporal changes in the low body weight status and the risk of fracture was stronger in older patients (> 65 years) than in young patients. Moreover, low body weight was also found to be substantially associated with osteoporosis in the elderly [39]. In addition, older persons are more prone to fall-related injuries, such as hip fractures, due to disorders such as muscular weakness or decreased eyesight and balance [40]. In our study, the correlation between fracture risk and low body weight change was greater in men than in women. According to a recent large-scale cohort study conducted in Norway, men with BMI < 22 kg/m^2^ had a greater risk of fracture than women [41, 42]. In this study, patients with hypertension or CKD also had a higher risk of fracture. High blood pressure and CKD can cause continuous calcium loss in urine, thereby hastening the mineral loss [43–45].

The significant strength of this study is its utilization of a nationally representative database from the general population. In addition, we examined the connection between temporal changes in the low body weight status and the risk of fracture by analyzing data from two consecutive national health examinations. Even if adults are classified as having low body weight at one point in time, our findings indicate that the risk is not constant and may fluctuate depending on changes in BMI over 2 consecutive years. This also demonstrates that even if the patient has a normal weight, they may be at a higher risk if they have low body weight in the future. This may allow physicians to assess and monitor patients more accurately. To the best of our knowledge, no prior studies have reported a correlation between temporal changes in the low body weight status and the risk of fracture.

This study has some limitations. First, we were unable to directly confirm the BMD T-scores. Low body weight influenced the BMD score, although the specific effect was unknown at the time of this study. Second, it is difficult to determine the specific number of fractures. This is because most cases of hip, wrist, and other fractures are diagnosed in hospitals, whereas vertebral fractures are often asymptomatic. To be as precise as possible, we used the operational definitions used in several previous studies [5, 23, 24]. Validation studies are the best techniques for confirming the given algorithm for diagnostic codes. The same operational criteria developed in earlier studies were applied to identify fractures in this analysis [5, 23, 24]. Furthermore, to detect fractures as precisely as possible, we excluded patients with a history of fractures and used a 1-year lag period after confirming the low body weight status. Due to the use of conservative methods in this study, it is possible that the incidence rate of fractures was greatly underestimated. Third, this study used a large nationwide population database, which limited the availability of detailed information on potential confounding factors such as medication history, cumulative exposure dosage (e.g., alcohol or nicotine), nutritional status (e.g. intake of macro/micronutrients and supplements) and detailed laboratory test result (e.g. sex hormones, growth hormones, cortisols). Although we adjusted for important covariates such as age, sex, smoking status, alcohol consumption, exercise habits, and comorbidities such as hypertension and chronic kidney disease in our analysis, residual confounding may still exist. Finally, this study used a national database from a single country's national health insurance system, making it impossible to adapt to multiple ethnic groups.

## Conclusion

Using a nationwide population cohort, this study evaluated whether low body weight is a significant risk factor for fracture in the Korean population aged > 40 years. Adults with low body weight aged over 40 years had a higher risk of fractures, even if they regained their normal weight. In particular, having a low body weight from being non-low body weight increased the chance of fractures the highest, followed by consistently low body weight. In addition, the risk of fractures was affected by age over 65 years, the male sex, hypertension, and CKD.

## Supplementary Information

**Additional file 1.** Definitions of covariates and measurements.

## Data Availability

The data that support the findings of this study are available from Korea National Health Insurance Service, but restrictions apply to the availability of these data, which were used under license for the current study, and so are not publicly available. Data are however available from the authors upon reasonable request and with permission of Korea National Health Insurance.
